# Morphological and Morphometric Characteristics of Anterior Maxilla Accessory Canals and Relationship with Nasopalatine Canal Type—A CBCT Study

**DOI:** 10.3390/diagnostics11081510

**Published:** 2021-08-21

**Authors:** Milica Vasiljevic, Pavle Milanovic, Nemanja Jovicic, Miroslav Vasovic, Dragan Milovanovic, Radisa Vojinovic, Dragica Selakovic, Gvozden Rosic

**Affiliations:** 1 Department of Dentistry, Faculty of Medical Sciences, University of Kragujevac, 34000 Kragujevac, Serbia; milicavaska13@gmail.com (M.V.); pavle11@yahoo.com (P.M.); miki_vasovic@yahoo.com (M.V.); 2 Department of Histology and Embryology, Faculty of Medical Sciences, University of Kragujevac, 34000 Kragujevac, Serbia; nemanjajovicic.kg@gmail.com; 3 Clinical Pharmacology Department, Clinical Centre Kragujevac, 34000 Kragujevac, Serbia; piki@medf.kg.ac.rs; 4 Department of Pharmacology and Toxicology, Faculty of Medical Sciences, University of Kragujevac, 34000 Kragujevac, Serbia; 5 Department of Radiology, Faculty of Medical Sciences, University of Kragujevac, 34000 Kragujevac, Serbia; rhvojinovic@gmail.com; 6 Department of Physiology, Faculty of Medical Sciences, University of Kragujevac, 34000 Kragujevac, Serbia

**Keywords:** accessory canal—AC, nasopalatine canal—NPC, anterior maxilla, cone beam computed tomography—CBCT, morphometric analysis

## Abstract

This study aimed to evaluate principal morphological and morphometric characteristics of accessory canals (ACs) of the anterior maxilla, as well as to analyze the relationship with nasopalatine canal (NPC) type. The results of our study showed that ACs were observed in almost 50% of participants. They were mostly presented bilaterally and in a curved shape, with a palatal foramen position. The morphometric characteristics of ACs were significantly influenced by NPC type. NPC type had the strongest impact on the distance between the NPC and AC, as well as on the distance between the AC and the facial aspect of buccal bone wall, in inferior parts of the alveolar ridge. On the other hand, the distance between the AC and central incisors was not significantly influenced by NPC shape in the lower region of the anterior maxilla. However, the participants with the banana-type of the NPC expressed the reduction in distance from the AC to the central incisor at the upper part in comparison with the subjects with the cylindrical-type of the NPC. On the basis of the results of this study, the simultaneous estimation of ACs and the NPC seems reasonable, as this approach may be useful in the prevention of complications which could occur during implant surgery interventions.

## 1. Introduction

The anterior maxilla is a region of interest for clinicians who perform various surgical interventions in oral and maxillofacial surgery, such as dental implant placement, cyst enucleation, orthognathic surgery, and the surgical removal of impacted and supernumerary teeth [1]. The knowledge of morphometric characteristics of this area seems to be crucial for the general success of therapeutic protocols, including the avoidance of neurovascular complications [2]. Numerous studies have shown the importance of the morphological estimation of the nasopalatine canal (NPC), as the most prominent anatomical structure in the anterior maxilla, which is helpful for clinicians during the planning of dental implant placement [3,4,5]. In addition to advanced diagnostic imaging in dentistry, the presence of accessory canals (ACs) is often neglected or misdiagnosed [6], and the damage of these structures during the implant placement may cause complications such as non-integration of a dental implant, mucosa necrosis, pain, paresthesia, hemorrhage, sensation of burning head in the occipital region, and neuropathy [7,8,9,10]. The majority of these symptoms could be linked to disturbance to the neurovascular bundle in ACs. 

One of ACs in anterior maxilla, *canalis sinuosus* (CS) [11], originates from the infraorbital canal and carries the anterior superior alveolar nerve and vessels [12,13]. According to ACs’ direction, von Arx and coworkers classified ACs into three types: curved, vertical, and Y-shaped [6]. The AC with a curved shape originates from CS and runs in a curved direction toward the alveolar process. The AC with a vertical shape has a vertical direction from the medial aspect of the nasal floor toward the alveolar ridge, while the Y-shaped AC represents communication between the CS branch and the branch from the medial aspect of the nasal floor [6]. ACs’ openings are in the alveolar ridge, and Oliveira-Santos and colleagues defined seven groups of localization of additional foramina (central incisors region between central and lateral incisors, lateral incisor region, canine region, first premolar region, and adjacent to incisive foramen, posterior, anterior, or lateral) [14]. On the other hand, Machado and coworkers classified terminal portions of ACs into three main groups: palatal, transversal, and buccal [15]. According to this classification, the predilection of AC foramen localization is palatal to the central incisors in approximately 57% [6]. 

While conventional radiographs have multiple visual limitations [16] which could result in a wrong display of anatomical configurations [17], the structures such as ACs could stay unnoticed. A significant improvement was achieved by using cone beam computed tomography (CBCT). Therefore, the disadvantages of conventional diagnostics, such as diameters smaller than 1 mm, a porous cortical layer, and a variable course, could complicate ACs’ examination [18]. On the other hand, the advantages of CBCT, such as its high resolution, cross-sectional view, and diagnostic reliability at lower costs and radiation, provide more accurate estimation of the anatomical variations in that region, including the better insight of ACs’ characteristics [19]. However, due to the unquestionable importance of CBCT slice thickness, it should be noted that for better visualization of ACs, Anatoly and coworkers advised the optimal slice thickness of 0.5 and 1 mm [12].

Morphological and morphometric characteristics of ACs as well as their relationship with the surrounding anatomical structures have not yet been fully evaluated in the literature. Although these anatomical structures have been described for more than 80 years [20], the current literature still reports clinical studies in which they are damaged during dental implant placement [10,21].

The aim of this study was to evaluate principal morphological and morphometric characteristics of ACs. In addition, since the NPC and ACs are considered to be the two most vulnerable structures in the anterior maxilla in the context of oral surgery interventions, we also estimated the impact of NPC type on ACs’ positioning in order to inform possible relationships with potential clinical importance.

## 2. Materials and Methods

### 2.1. Patients’ Selection

The source of data for this retrospective CBCT study was from radiological databases of patients who came to the Department of Dentistry, Faculty of Medical Sciences, University of Kragujevac, Serbia, in the period between November 2020 and April 2021. This research was approved by the institutional review board of the Faculty of Medical Sciences, University of Kragujevac (identification code of approval 01-4376, issued on 15 April 2021). The inclusion criteria were the following: ≥18 years old, presence of both central maxillary incisors, and written informed consent from patients. The exclusion criteria for participants included subjects with a traumatic and pathological lesion in the anterior maxilla, such as a nasopalatine duct cyst, a periapical lesion, tumors, and cleft lips. Patients with previous surgical procedures in this region, dental implants, bone grafting, and subjects with orthodontic braces and metal restoration were also excluded. Low-quality images with technical artefacts were excluded from the research as well. Following the predefined criteria, a total of 130 subjects were included in this study (69 male and 61 female, with an average age of 45.1 ± 1.98 and 41.25 ± 1.72, respectively).

### 2.2. Characteristics of Imaging Device and Software for Evaluation of Images

The Orthophos XG 3D device (Sirona Dental Systems GmbH, Bensheim, Germany), with three-dimensional settings for recording, VOL1 HD (85 kV/6 mA, exposure time—14.3 s) or VOL2 HD (85 kV/10 mA, exposure time—5.0 s), and a voxel size of 160 μm or 100 μm, respectively, were used for obtaining the scans. All CBCT images had the field of view 8 × 8 cm. 

The images were analyzed by using GALAXIS software v1.9.4 (Sirona Dental Systems GmbH, Bensheim, Germany) in sagittal, coronal, and axial view with a slice thickness of 0.5 mm. The examination was conducted by using a 23-inch Philips LED monitor with a resolution of 1920 × 1080 pixels, in a room with dim lighting. Brightness and contrast were adjusted by using software settings.

### 2.3. NPC and ACs’ Evaluation of CBCT Images

**Figure 1:**
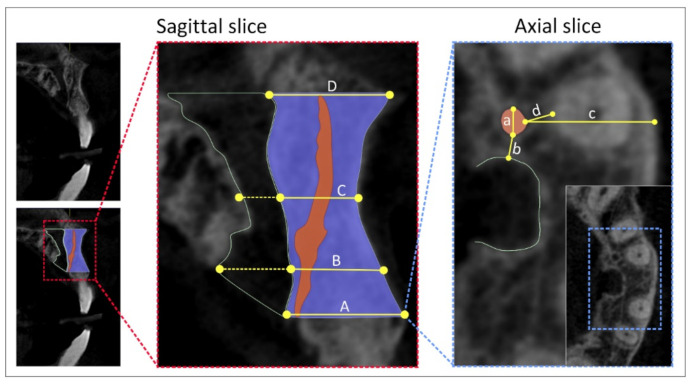
CBCT slices and landmarks of interest. Sagittal cross-section—sagittal CBCT slice (**left**, **upper**); slice with marked field of interest (**left**, **bellow**); selected landmarks for analyses (**right**)—(A) the distance between the buccal border of incisive foramen and facial aspect of the buccal bone wall, (B) the distance between the buccal wall of the nasopalatine canal and facial aspect of the buccal bone wall using a horizontal line from the palatal border of the incisive foramen, (C) the distance between the buccal border at the midpoint level of NPC length and facial aspect of the buccal bone wall, (D) the distance between the buccal border of nasal foramen and facial aspect of the buccal bone wall; Axial cross-section—axial CBCT slice (**right**); (a) the diameter of AC, (b) the distance between AC and NPC, (c) the distance between AC and facial aspect of the buccal bone wall, (d) the distance between AC and central incisor root.

**Figure 2:**
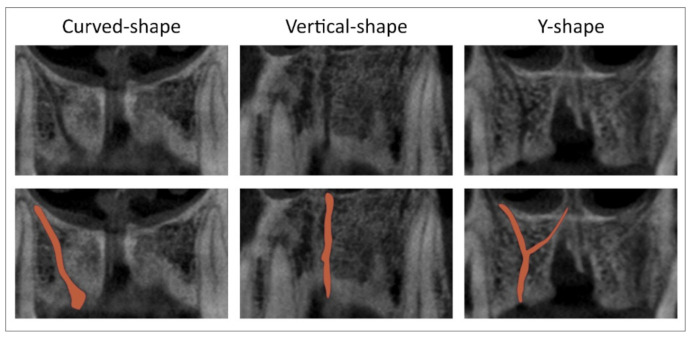
CBCT slices and marks of interest. Accessory canal shapes at the coronal cross-section (**upper**); red marks define ACs shapes at the coronal cross-section (**below**).

Using a sagittal slice, we estimated four types of NPC shapes following the previously defined classification [22], at predefined anterior maxilla levels (Figure 1) as recently described [5]. ACs’ evaluation was performed by using sagittal, coronal, and axial slices, as follows:

1. sagittal slices were used for the evaluation of ACs’ presence in the maxillary central incisor area;
2. coronal slices were used for ACs’ direction determination (vertical-, curved-, and Y-shaped) as shown in Figure 2;
3. axial slices were used for ACs’ position determination in maxillary central incisors’ area, as well as for the quantification of the following parameters: ACs’ diameter, the distance between the AC and NPC, the distance between the AC and the central incisor, and the distance between the AC and the facial aspect of the buccal bone wall.

All parameters were obtained at four predefined levels, and expressed in mm. Two independent observers estimated all parameters, with demonstrated acceptable rater reliability and the level of agreement, and the mean value for each parameter was taken for further evaluation.

### 2.4. Statistical Analysis

The data presented herein were expressed as the means ± SEM. The parameters were initially submitted to Levene’s test for homogeneity of variance and to the Shapiro–Wilk test of normality. The comparisons between the groups were performed using the Chi-square test or one-way ANOVA, followed by Scheffe’s post hoc test. A *p* value < 0.05 was considered significant. Statistical analysis was performed with the SPSS version 20.0 statistical package (IBM SPSS Statistics 20, Armonk, NY, USA).

## 3. Results

The presence of an accessory canal in the anterior maxilla at the level of the central incisors was confirmed in slightly below a half of the total number of participants (Table 1). Although the appearance of ACs in male subjects was discretely above 50% and in female participants below the same percentage, it was not confirmed that sex of the participants had a statistically significant impact. Similarly, the estimation of the influence of age on the occurrence of ACs showed no statistical significance (Table 1). 

As presented in Table 2, the analysis of ACs’ localization distribution showed variations in distribution. Namely, while single-side presentation was more prominent on the left, the bilateral appearance of ACs was confirmed in the majority of subjects. This algorithm was not significantly affected by sex nor by the age of participants.

ACs in the anterior maxilla at the level of the central incisors, as shown in Table 3, were classified according to the canal shape into three categories: vertical, curved, and Y-shaped. Statistical analysis confirmed the predominance of the curved shape (almost 50%), while the Y shape was present in only 15% of participants. Similarly to the AC localization, there was no significant impact of either sex or age on the AC shape.

The presentation of AC foramen localization, as shown in Table 4, was predominantly shifted to palatal (more than 80%), while transversal foramen localization was observed in approximately 15% of subjects. It is worth noting that, although described in the literature, there was no buccal localization of the AC foramen observed in this study. The dominant appearance of the AC foramen in the palatal position was obvious in both male and female participants. Interestingly, the transversal localization of the AC foramen was significantly reduced in older subjects (Table 4).

**Figure 3:**
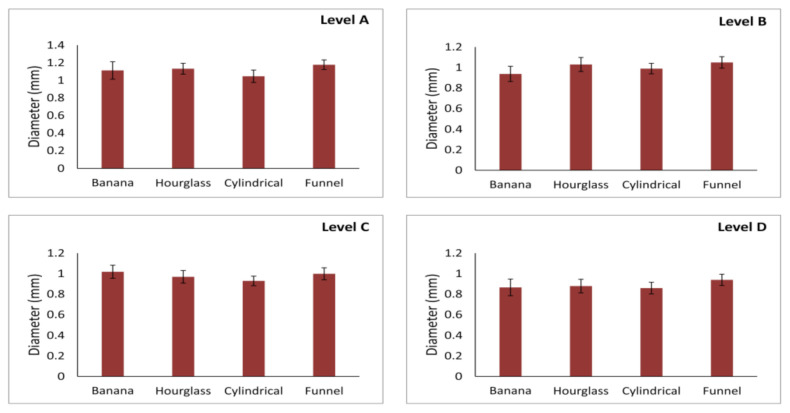
Maximal diameter of accessory canal at different levels of anterior maxilla according to NPC shape. Values are expressed as the mean ± SEM.

As shown in Figure 3, the maximal diameter of the AC was not significantly affected by NPC shape at all (A, B, C, and D) estimated levels (df = 3, F = 0.738, 0.498, 0.352, and 0.329, respectively). The mean maximal AC diameter was approximately 1 mm for all levels. Although the maximal diameter was slightly above the mean value on the level A, while on the other hand the lowest maximal diameter was observed at the level D, there was no significant difference for this parameter.

**Figure 4:**
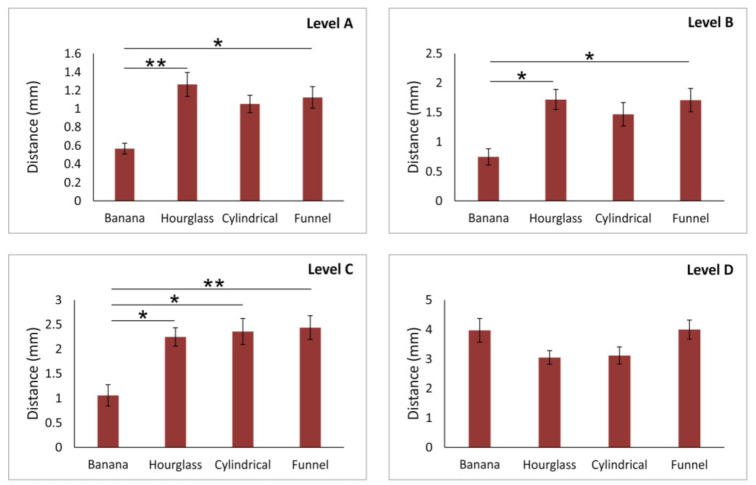
The distance of accessory canal from NPC at different levels of anterior maxilla according to NPC shape. Values are expressed as the mean ± SEM. * denotes a significant difference of *p* < 0.05, ** denotes a significant difference of *p* < 0.01.

However, the analysis presented in Figure 4 confirmed that the distance of the AC from the NPC was significantly influenced by NPC shape, except on the level D (df = 3, F = 2.946). Therefore, the impact of NPC shape on AC distance from the NPC was confirmed at the levels A, B, and C (F = 4.507, 3.717, and 4.617, respectively) The distance between the AC and NPC in the banana-type NPC was significantly lower in comparison to both hourglass- (*p* < 0.01 at level A, and 0.05 at level B) and funnel-type (*p* < 0.01 at both levels A and B). The most robust impact of NPC shape on AC-NPC distance was present at the level C where the distance from the NPC observed in the banana-type was also significantly reduced when compared to the cylindrical-type of NPC (*p* < 0.05).

**Figure 5:**
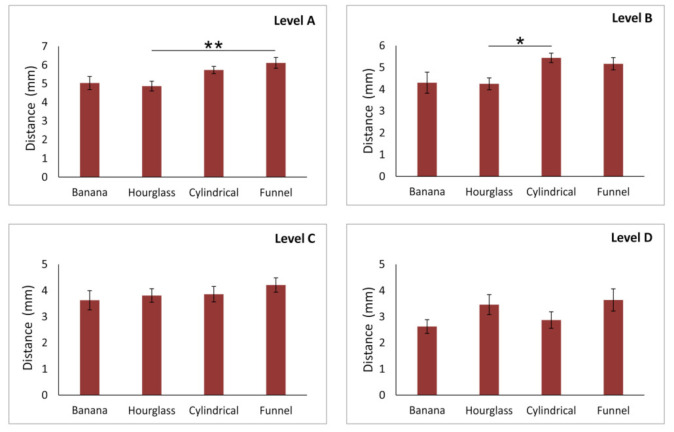
The distance of accessory canal from facial aspect of the buccal bone wall at different levels of anterior maxilla according to NPC shape. Values are expressed as the mean ± SEM. * denotes a significant difference of *p* < 0.05, ** denotes a significant difference of *p* < 0.01.

As presented in Figure 5, the distance of the AC from the facial aspect of the buccal bone wall was significantly altered according to NPC shape at the lower part of the alveolar ridge (level A and B, F = 4.840 and 4.253, respectively). The distance between the AC and the cortical layer was significantly reduced at both estimated levels in subjects with the hourglass-type compared to the funnel-type of NPC (*p* < 0.01 at the level A, *p* < 0.05 at the level B). At the same time, there was no significant impact of NPC shape on the distance AC from the facial aspect of the buccal bone wall in the upper region of the anterior maxilla (level C and D, F = 0.656 and 1.348, respectively).

**Figure 6:**
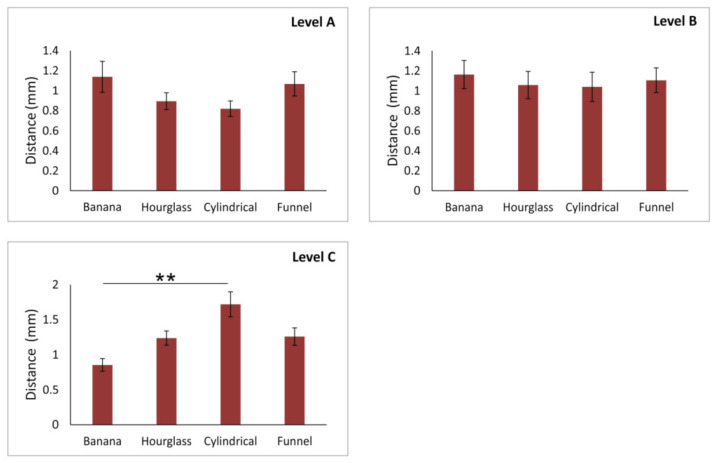
The distance of accessory canal from central incisor at different levels of anterior maxilla according to NPC shape. Values are expressed as the mean ± SEM. ** denotes a significant difference of *p* < 0.01.

In contrast, the distance between the AC and central incisors (Figure 6) was not significantly influenced by NPC shape in the lower region of the anterior maxilla (level A and B, F = 1.707 and 1.109, respectively), but at the upper part of the alveolar bone (level C, F = 5.258), the participants with the banana-type of the NPC expressed the reduction in distance from the AC to the central incisor when compared to subjects with the cylindrical-type of NPC (*p* < 0.01). Obviously, the statistical analysis for the distance of the AC from the central incisor at the level D was not performed due to insufficient data (this parameter was quantified in only eight subjects).

## 4. Discussion

The aim of this study was to present the basic information considering morphologic and morphometric characteristics of ACs in the anterior maxilla. Furthermore, we intended to offer the estimation of spatial relationships in the region of interest for a number of surgical interventions, such as the distances between ACs and the NPC, central incisor, and facial aspect of the buccal bone wall. Most of the previous studies analyzed the NPC as the main neurovascular anatomical structure in the anterior maxilla, which could interfere with the maxillary central incisor implant placement [5,6,23]. Although the clinical importance of ACs was confirmed in literature data [10,24,25], the specific characteristics of this structure, in the context of its relevance in surgical interventions, had not been sufficiently explored before.

In order to provide a better insight into neurovascular distribution that depends on ACs’ characteristics, having in mind that this may seriously affect the postoperative outcome of dental implant placements in the anterior maxilla, we presented data that could provide observed algorithms of potential clinical importance. 

Previous studies reported the prevalence of ACs with a very extensive range, from 16% [14] to 100% [26]. In this study, ACs were observed in approximately 50% which is in accordance with Machado et al. [15] and von Arx and coworkers [6]. However, Wanzeler and colleagues reported the prevalence of ACs in approximately 90% [27]. These differences could be attributed to the diversity of methodological, ethnical, and racial characteristics [27]. Furthermore, no impact of sex to ACs’ prevalence was confirmed in this investigation, which is in accordance with previous reports [6,14,26]. Nevertheless, it should be noted that literature data allow that the ACs’ prevalence was significantly higher in both male [15,28] and female subjects [12]. Likewise, the presence of ACs was also not altered by age, which is in line with previous investigations [6,29]. 

Interestingly, the bilateral presence of ACs was observed in 50 % of participants, while AC unilateral presentation was confirmed almost equally on the left and right sides. This is in accordance with the study conducted by Anatoly [12] and Aoki and coworkers [25]. On the other hand, ACs’ distribution reported by Manhaes showed a significant prevalence on the left side, with only a few bilateral localizations [30]. 

Applying the ACs shape classification established by von Arx and coworkers [6], we found a significant difference in ACs’ shape distribution (Table 3), with the prevalence of a curved shape, followed by a vertical and Y shape of ACs (48.96 %, 36.45 %, and 14.58 %, respectively). The distribution of ACs’ shape observed in this study is in line with the previous investigations [6,28]. Following the previous studies [6,15,31,32] that described the importance of ACs’ foramen localization as one of the risk factors for neurovascular implant-related injury in the central incisors area, we focused on this specific region. Our study confirmed the existence of a significant difference in AC foramen localization incidence depending on the predefined criteria [15], as follows: the most frequent localization was palatal (83.33 %), followed by transversal (16.66 %), while buccal localization was not observed. This distribution is in accordance with the results of the study by Machado and coworkers [15]. 

Implant surgery requires detailed information about neurovascular-containing structures, such as ACs, in order to avoid severe complications. For this reason, we evaluated ACs’ morphometric characteristics at four (consecutive) levels since it is not a standard methodological approach that is principally based on the analysis undertaken on one level [6,14]. Our results showed that ACs’ diameter remained almost constant, at all levels (approximately 1 mm), with a slight decrease from the caudal to cranial part. Those results are in line with Machado and coworkers [15], while, on the other hand, Oliveira Santos and collaborators found a slightly larger (1.4 mm) ACs’ diameter [14]. The information about the observed mean diameters of ACs should be considered as important, since literature data described extensive hemorrhage caused by iatrogenic injury (during dental implant placement) of blood vessels which are less than 1 mm in diameter [33]. Summarizing the results regarding the morphological and morphometric characteristics of ACs, it can be described as an anatomical structure represented in 50% of cases with predominant bilateral and palatal localization, curved shape, and about 1 mm diameter at all predefined levels which is in line with previous studies [6,12,15,25]

Periapical inflammation and previous dentoalveolar injuries can affect dimensions of anatomical structures in the anterior maxilla, as well as their radiographic presentation, which emphasizes the need for detailed preoperative 3D analysis [34]. Numerous studies described that the lack of ACs’ position determination may lead to diagnostic confusion considering the misdiagnosis for periapical lesions instead of injury of neurovascular elements of ACs [35,36], with certain postoperative complications [7,18]. In their study, Rosano and coworkers described the lesion of CS after implant placement, although precise digital planning has been performed [10]

NPC, as the most prominent structure in the premaxilla, has a confirmed importance in planning oral surgery interventions (5). So, the key point of the present in this study was to analyze the interconnection between NPC shape and ACs’ characteristics, as the two most vulnerable structures in the anterior maxilla (8). Our results clearly imply that NPC type had the strongest impact on the distance between the NPC and AC in inferior parts of the alveolar ridge (Figure 4). Namely, we observed the smallest distance between the AC and NPC in subjects with the banana-type NPC at A, B, and C levels. In contrast, the participants with the hourglass-type NPC expressed the largest distance at the same (lower) parts of the anterior maxilla. This algorithm may be important especially in the cases where implant artifacts may affect the visualization of ACs [7].

In order to complete ACs’ localization analysis, we also estimated the distance between the facial aspect of the buccal bone wall and the AC according to the NPC shape. Those results also confirmed a significant impact of NPC type on the distance between the AC and the facial aspect of the buccal bone wall in inferior parts of the alveolar ridge. Following the observed iterations for the distance between the NPC and AC, as well as between the buccal bone wall and the AC, some potential anticipation for AC localization could be achieved in the following way: subjects with the hourglass-type NPC have the largest distance between the NPC and AC, and the smallest distance between the facial aspect of the buccal bone wall and the AC at A and B levels, so it could be expected that the AC is located closer to the buccal side.

Alkanderi and collaborators presented the data that the smallest distance between the central incisor root and the NPC significantly increased the NPC perforation rate [4], so the recommendation for immediate implant placement, in the anterior maxilla, is a more palatal direction to achieve good aesthetics and primary implant stability [37]. However, this approach may affect the surrounding ACs. According to the results of this study, the central incisor in subjects with the banana-type NPC was significantly closer to the AC, while subjects with the cylindrical-type NPC expressed the more prominent distance. Following the observed algorithm, it could be predicted that the highest rate of potential AC perforations in the subjects with the banana-type of NPC could occur at the level C.

Practitioners should be fully aware of ACs’ presence and their morphological characteristics and take it into account during dental implant planning. In modern oral and maxillofacial surgery, CBCT is one of the best diagnostic tools in the diagnosis of various pathological conditions. Furthermore, it provides an insight into various anatomical structures of interest to clinicians, especially implantologists, as its appearance could not be analyzed using conventional radiographs [38,39]. Using CBCT, however, it is possible to observe a region of interest in all three dimensions, which allows a precise evaluation of smaller anatomical structures such as ACs in the anterior maxilla [10]. Therefore, the American Academy of Oral and Maxillofacial Radiology recommends the use of CBCT as the method of choice for preoperative implant placement planning to assess the location of a future implant [40]. Moreover, CBCT software allows for virtual implant placement to determine the appropriate characteristics of the implant (size, shape, position, etc.) and to avoid complications that may arise due to inadequate surgical intervention planning [41,42].

## 5. Conclusions

In summary, the results of our study showed that ACs in the anterior maxilla are present with a high prevalence and that their morphological characteristics may be assessed using CBCT. They are mostly presented bilaterally and in a curved shape, with a palatal foramen position. The algorithms of interconnection between ACs and surrounding anatomical structures may be a potential clinically relevant checkpoint in the planning of dental implant placement in the anterior maxilla. Moreover, the simultaneous estimation of the NPC and ACs, as two of the most vulnerable structures in the anterior maxilla, may be useful in the prevention of complications which may occur during implant surgery interventions.

## Figures and Tables

**Table 1:** Data for presence of accessory canal in anterior maxilla at the level of central incisors.

**Presence** [0.031, 1, 0.861]	64/130 (49.23%)					
**Sex** [0.278, 1, 0.598]	**Male**			**Female**		
	Yes—36	No—33		Yes—28	No—32	
**Years of age** [2.022, 2, 0.364]	**18−37**		**38−57**		**58−80**	
	Yes—26	No—19	Yes—27	No—29	Yes—12	No—17

Values in square brackets denote Chi-Square, df, and *p*, respectively.

**Table 2:** Accessory canal localization.

**Localization** * [8.844, 2, 0.012]	**Left Side Only**—19		**Right Side Only**—13		**Bilateral**—32	
**Sex** [0.051, 2, 0.975]	Male	11	Male	7	Male	18
	Female	8	Female	6	Female	14
**Years of age** [6.293, 4, 0.178]	18−37	8	18−37	2	18−37	15
	38−57	9	38−57	6	38−57	12
	58−80	2	58−80	5	58−80	5

Values in square brackets denote Chi-Square, df, and *p*, respectively; * denotes significance <0.05.

**Table 3:** Accessory canal localization.

**Canal Shape** ** [17.438, 2, 0.000]	**Vertical Shape**—35		**Curved Shape**—47		**Y Shape**—14	
**Sex** [3.344, 2, 0.188]	Male	18	Male	25	Male	11
	Female	17	Female	22	Female	3
**Years of age** [2.763, 4]	18−37	17	18−37	19	18−37	4
	38−57	13	38−57	18	38−57	8
	58−80	5	58−80	10	58−80	2

Values in square brackets denote Chi-Square, df, and *p*, respectively; ** denotes significance <0.01.

**Table 4:** Accessory canal foramen localization.

**Foramen Localization** ** [42.667, 1, 0.000]	**Palatal**—80		**Transversal**—16		**Buccal**—0	
**Sex** [0.305, 1, 0.581]	Male	44	Male	10	Male	0
	Female	36	Female	6	Female	0
**Years of age** ** [97.374, 4, 0.000]	18−37	30	18−37	10	18−37	0
	38−57	33	38−57	6	38−57	0
	58−80	17	58−80	0	58−80	0

Values in square brackets denote Chi-Square, df, and *p*, respectively; ** denotes significance <0.01.

## Data Availability

All data available upon request.

## References

[1] Den Hartog L., Slater J.J., Vissink A., Meijer H.J., Raghoebar G.M. (2008). Treatment outcome of immediate, early and conventional single-tooth implants in the aesthetic zone: A systematic review to survival, bone level, soft-tissue, aesthetics and patient satisfaction. J. Clin. Periodontol..

[2] Arruda J.A., Silva P., Silva L., Álvares P., Silva L., Zavanelli R., Rodrigues C., Gerbi M., Sobral A.P., Silveira M. (2017). Dental Implant in the Canalis Sinuosus: A Case Report and Review of the Literature. Case Rep. Dent..

[3] Bornstein M.M., Balsiger R., Sendi P., von Arx T. (2011). Morphology of the nasopalatine canal and dental implant surgery: A radiographic analysis of 100 consecutive patients using limited cone-beam computed tomography. Clin. Oral Implants Res..

[4] Alkanderi A., Al Sakka Y., Koticha T., Li J., Masood F., Suárez-López Del Amo F. (2020). Incidence of nasopalatine canal perforation in relation to virtual implant placement: A cone beam computed tomography study. Clin. Implant Dent. Relat. Res..

[5] Milanovic P., Selakovic D., Vasiljevic M., Jovicic N.U., Milovanović D., Vasovic M., Rosic G. (2021). Morphological Characteristics of the Nasopalatine Canal and the Relationship with the Anterior Maxillary Bone-A Cone Beam Computed Tomography Study. Diagnostics.

[6] Von Arx T., Lozanoff S., Sendi P., Bornstein M.M. (2013). Assessment of bone channels other than the nasopalatine canal in the anterior maxilla using limited cone beam computed tomography. Surg. Radiol. Anat..

[7] Volberg R., Mordanov O. (2019). Canalis Sinuosus Damage after Immediate Dental Implant Placement in the Esthetic Zone. Case Rep. Dent..

[8] McCrea S.J.J. (2017). Aberrations Causing Neurovascular Damage in the Anterior Maxilla during Dental Implant Placement. Case Rep. Dent..

[9] Neves F.S., Crusoé-Souza M., Franco L.C., Caria P.H., Bonfim-Almeida P., Crusoé-Rebello I. (2012). Canalis sinuosus: A rare anatomical variation. Surg. Radiol. Anat..

[10] Rosano G., Testori T., Clauser T., Massimo Del Fabbro M. (2021). Management of a neurological lesion involving CanalisSinuosus: A case report. Clin. Implant Dent. Relat. Res..

[11] Jones F.W. (1939). The anterior superior alveolar nerve and vessels. J. Anat..

[12] Anatoly A., Sedov Y., Gvozdikova E., Mordanov O., Kruchinina L., Avanesov K., Vinogradova A., Golub S., Khaydar D., Hoang N.G. (2019). Radiological and Morphometric Features of Canalis Sinuosus in Russian Population: Cone-Beam Computed Tomography Study. Int. J. Dent..

[13] Heasman P.A. (1984). Clinical anatomy of the superior alveolar nerves. Brit. J. Oral. Maxillofac. Surg..

[14] De Oliveira-Santos C., Rubira-Bullen I.R., Monteiro S.A., León J.E., Jacobs R. (2013). Neurovascular anatomical variations in the anterior palate observed on CBCT images. Clin. Oral Implants Res..

[15] Machado V.C., Chrcanovic B.R., Felippe M.B., Manhães Júnior L.R., de Carvalho P.S. (2016). Assessment of accessory canals of the canalis sinuosus: A study of 1000 cone beam computed tomography examinations. Int. J. Oral. Maxillofac. Surg..

[16] Baciut M., Hedesiu M., Bran S., Jacobs R., Nackaerts O., Baciut G. (2013). Pre- and postoperative assessment of sinus grafting procedures using cone-beam computed tomography compared with panoramic radiographs. Clin. Oral Implants Res..

[17] Temmerman A., Hertelé S., Teughels W., Dekeyser C., Jacobs R., Quirynen M. (2011). Are panoramic images reliable in planning sinus augmentation procedures?. Clin. Oral Implants Res..

[18] Shah P.N., Arora A.V., Kapoor S.V. (2017). Accessory branch of canalis sinuosus mimicking external root resorption: A diagnostic dilemma. J. Conserv. Dent..

[19] Allareddy V., Vincent S.D., Hellstein J.W., Qian F., Smoker W.R., Ruprecht A. (2012). Incidental findings on cone beam computed tomography images. Int. J. Dent..

[20] Lopes Dos Santos G., Ikuta C.R.S., Salzedas L.M.P., Miyahara G.I., Tjioe K.C. (2020). Canalis sinuosus: An Anatomic Repair that May Prevent Success of Dental Implants in Anterior Maxilla. J. Prosthodont..

[21] Shintaku W.H., Ferreira C.F., Venturin J.S. (2020). Invasion of the canalis sinuosus by dental implants: A report of 3 cases. Imaging Sci. Dent..

[22] Mardinger O., Namani-Sadan N., Chaushu G., Schwartz-Arad D. (2008). Morphologic Changes of the Nasopalatine Canal Related to Dental Implantation: A Radiologic Study in Different Degrees of Absorbed Maxillae. J. Periodontol..

[23] Jia X., Hu W., Meng H. (2015). Relationship of central incisor implant placement to the ridge configuration anterior to the nasopalatine canal in dentate and partially edentulous individuals: A comparative study. PeerJ.

[24] Shelley A., Tinning J., Yates J., Horner K. (2019). Potential neurovascular damage as a result of dental implant placement in the anterior maxilla. Br. Dent. J..

[25] Aoki R., Massuda M., Zenni L.T.V., Fernandes K.S. (2020). Canalis sinuosus: Anatomical variation or structure?. Surg. Radiol. Anat..

[26] Baena-Caldas G., Rengifo-Miranda H., Heerera-Rubio A., Peckham X., Zúñiga J. (2019). Frequency of canalis sinuosus and its anatomic variations in cone beam computed tomography images. Int. J. Morphol..

[27] Wanzeler A.M., Marinho C.G., Alves Junior S.M., Manzi F.R., Tuji F.M. (2015). Anatomical study of the canalis sinuosus in 100 cone beam computed tomography examinations. Oral Maxillofac. Surg..

[28] Tomrukçu D.N., Köse T.E. (2020). Assesment of accessory branches of canalis sinuosus on CBCT images. Med. Oral Patol. Oral Cir. Bucal.

[29] Shan T., Qu Y., Huang X., Gu L. Cone beam computed tomography analysis of accessory canals of the canalis sinuosus: A prevalent but often overlooked anatomical variation in the anterior maxilla. J. Prosthet. Dent..

[30] Manhães Júnior L.R., Villaça-Carvalho M.F., Moraes M.E., Lopes S.L., Silva M.B., Junqueira J.L. (2016). Location and classification of Canalis sinuosus for cone beam computed tomography: Avoiding misdiagnosis. Braz. Oral Res..

[31] Orhan K., Gorurgoz C., Akyol M., Ozarslanturk S., Avsever H. (2018). An anatomical variant: Evaluation of accessory canals of the canalis sinuosus using cone beam computed tomography. Folia Morphol..

[32] Ghandourah A.O., Rashad A., Heiland M., Hamzi B.M., Friedrich R.E. (2017). Cone-beam tomographic analysis of canalis sinuosus accessory intraosseous canals in the maxilla. Ger. Med. Sci..

[33] Kalpidis C.D., Setayesh R.M. (2004). Hemorrhaging associated with endosseous implant placement in the anterior mandible: A review of the literature. J. Periodontol..

[34] Suter V.G., Jacobs R., Brücker M.R., Furher A., Frank J., von Arx T., Bornstein M.M. (2016). Evaluation of a possible association between a history of dentoalveolar injury and the shape and size of the nasopalatine canal. Cli. Oral Investig..

[35] Shelley A.M., Rushton V.E., Horner K. (1999). Canalis sinuosus mimicking a periapical inflammatory lesion. Br. Dent. J..

[36] Vâlcu M., Rusu M.C., Sendroiu V.M., Didilescu A.C. (2011). The lateral incisive canals of the adult hard palate—Aberrant anatomy of a minor form of clefting?. Rom. J. Morphol. Embryol..

[37] Chung S.H., Park Y.S., Chung S.H., Shon W.J. (2014). Determination of implant position for immediate implant placement in maxillary central incisors using palatal soft tissue landmarks. Int. J. Oral Maxillofac. Implants.

[38] Milanovic P., Vasiljevic M. (2021). Gender Differences in the Morphological Characteristics of the Nasopalatine Canal and the Anterior Maxillary Bone—CBCT Study. Serb. J. Exp. Clin. Res..

[39] Jacobs R., Salmon B., Codari M., Hassan B., Bornstein M.M. (2018). Cone beam computed tomography in implant den-tistry: Recommendations for clinical use. BMC Oral Health.

[40] Tyndall D.A., Price J.B., Tetradis S., Ganz S.D., Hildebolt C., Scarfe W.C. (2012). Position statement of the American Academy of Oral and Maxillofacial Radiology on selection criteria Sfor the use of radiology in dental implantology with emphasis on cone beam computed tomography. Oral Surg. Oral Med. Oral Pathol. Oral Radiol..

[41] Nickenig H.J., Eitner S. (2007). Reliability of implant placement after virtual planning of implant positions using cone beam CT data and surgical (guide) templates. J. Craniomaxillofac. Surg..

[42] Kernen F., Kramer J., Wanner L., Wismeijer D., Nelson K., Flügge T. (2020). A review of virtual planning software for guided implant surgery—Data import and visualization, drill guide design and manufacturing. BMC Oral Health.

